# Quality estimation of the electrocardiogram using cross-correlation among leads

**DOI:** 10.1186/s12938-015-0053-1

**Published:** 2015-06-20

**Authors:** Eduardo Morgado, Felipe Alonso-Atienza, Ricardo Santiago-Mozos, Óscar Barquero-Pérez, Ikaro Silva, Javier Ramos, Roger Mark

**Affiliations:** Department of Signal Theory and Communications, Rey Juan Carlos University, Camino del Molino s/n, 28943 Fuenlabrada, Madrid Spain; Laboratory for Computational Physiology, MIT, 77 Massachusetts Ave, Cambridge, 02139 MA USA

**Keywords:** Electrocardiography, Signal quality, e-Health, Tele-monitoring

## Abstract

**Background:**

Fast and accurate quality estimation of the electrocardiogram (ECG) signal is a relevant research topic that has attracted considerable interest in the scientific community, particularly due to its impact on tele-medicine monitoring systems, where the ECG is collected by untrained technicians. In recent years, a number of studies have addressed this topic, showing poor performance in discriminating between clinically acceptable and unacceptable ECG records.

**Methods:**

This paper presents a novel, simple and accurate algorithm to estimate the quality of the 12-lead ECG by exploiting the structure of the cross-covariance matrix among different leads. Ideally, ECG signals from different leads should be highly correlated since they capture the same electrical activation process of the heart. However, in the presence of noise or artifacts the covariance among these signals will be affected. Eigenvalues of the ECG signals covariance matrix are fed into three different supervised binary classifiers.

**Results and conclusion:**

The performance of these classifiers were evaluated using PhysioNet/CinC Challenge 2011 data. Our best quality classifier achieved an accuracy of 0.898 in the test set, while having a complexity well below the results of contestants who participated in the Challenge, thus making it suitable for implementation in current cellular devices.

## Background

The electrocardiogram (ECG) signal is a standard clinical tool for diagnosis and monitoring of cardio-electrical function. The ECG measures the electrical activity of the heart using different electrode lead configurations, placed on the body surface of the patient. Clinical interpretation of the ECG requires waveform data of high quality. However, ECG signals are commonly distorted by artifacts, both physiological (muscular activity, patient motion) and non-physiological (electromagnetic interference, cable and electrode malfunction) in nature [1]. Thus, automatic estimation of ECG quality is of paramount importance, particularly in tele-monitoring applications where the ECG is commonly collected by untrained or inexperienced technicians; or even self-monitoring applications, where the patient collects his ECG following some basic instructions. Tele-ECG applications will make a difference in developing countries lacking adequate primary care capacity. In such scenarios, automatic real-time assessment of ECG quality is required in order to alert the technician about the need to repeat the ECG while the patient is still present. This task could be performed by current cellular terminals (smartphones) able to capture and to instantaneously estimate the quality of the ECG [2].

Quality estimation of the ECG signals is a challenging problem, which has captured the attention of the scientific community. The PhysioNet/CinC Challenge 2011 was devoted to this issue [2], for which an extensive number of algorithms were developed for the competition [3–14] (see Table 1 for comparative performances). The Challenge was later followed by the special issue of Physiological Measurement, Volume 33, Number 9 [15], where some of the participants extended their work and results originally submitted to the Challenge [1, 16–20]. Most of the above mentioned studies aimed to classify the ECG quality as “acceptable” or “unacceptable” by using either machine learning techniques or threshold detectors (rule-based methods). From the machine learning approach, excellent classification results were obtained [4–6, 16]. Clifford et al. [4] proposed a support vector machine (SVM) classifier based on previously defined quality indexes [21] extracted from morphological, spectral and statistical features of the ECG. Using this approach Clifford et al. scored 0.926 on accuracy in event 1 of the Challenge. This method was further refined in [16] by incorporating an additional quality index, and balancing and relabelling the original data. With this approach Clifford et al. obtained an accuracy up to 0.970 in the test set. Kalkstein et al. [5] proposed a combination of KNN and random forest methods using 72 features calculated from the correlation between leads and the ECG signal energy, reaching a 0.912 accuracy in event 1. Zausender et al. [6] also used a random forest approach applied on simple spectral features, yielding an accuracy of 0.904 in event 1. Besides the challenge participants, Nasery et al. proposed an algorithm based on the energy, concavity and correlation of the ECG signals [22, 23]. The correlation is analyzed by comparing each ECG signal with an estimated version of it. The estimation of the ECG signal from one lead is obtained by fitting a Neural Network classifier trained with the remaining leads and its derivatives.

**Table 1:** Performance comparison of ECG signal quality algorithms

	E1	E2	E3
Physionet/CinC Challenge (accuracy scores)			
Xia et al. [3]^a^	0.932	0.914	0.845
Clifford et al. [4]	0.926	$-$	$-$
Tat et al. [7]	0.920	$-$	$-$
Hayn et al. [8, 17]	0.916	0.834	0.873
Kalkstein et al. [5]	0.912	$-$	$-$
Jekova et al. [9, 18]	0.908	$-$	$-$
Zausender et al. [6]	0.904	$-$	$-$
Noponen et al. [10]	0.900	$-$	$-$
Moody [11]	0.896	0.896	0.802
Johannesen et al. [13, 20]	0.880	0.880	0.791
Langley et al. [12]	0.868	0.868	0.814
Chudacek et al. [14]	0.828	0.833	0.872
Other studies (accuracy score in the test set^b^)			
Clifford et al. [16]	0.970		
Xia et al. [1]^c^	0.951		
Langley et al. [19]	0.914		

Physionet/CinC Challenge was divided into three events: event 1 (E1), where participants were not required to submit their code; event 2 (E2), where participant were required to submit the code; and event 3 (E3), where the open source code of E2 was tested on a data set not available for participants. Accuracy scores for both E1 and E2 are calculated on the Dataset B by the Challenge organizers (see "Methods" section), while for E3 the accuracy was calculated on Dataset C.

^a^The score reported in [3] is different from the official entry [2].

^b^The test set is different from the Physionet/Cinc Challenge set.

^c^The reported accuracy was calculated for the training set.

Rule-based methods also provided remarkable results [1, 3, 7, 8, 10–14, 17, 19, 20], being the set of computed ECG parameters the main difference among these studies. Xia et al. [1, 3] reached the highest score of the competition, 0.932 in event 1. They combined different features, such us flat baseline detection, missing lead identification, and auto and cross correlation among ECG signal leads. Tat et al. [7] scored 0.92 in event 1 by combining QRS parameters, flat line detection, noise detection and ECG amplitude distribution measurements. Hayn et al. [8, 17] used basic signal properties (amplitude, saturation and flat baseline), number of crossing points between leads, and QRS quality metrics. Hayn et al. scored 0.916 in event 1 and 0.873 (1st place) in event 3. Jekova et al. [9, 18] proposed an algorithm based on scoring the noise level by analyzing the ECG amplitude and slopes in different frequency bands. They attained a score of 0.908 in event 1. Noponen et al. [10] estimated each lead signal as a linear combination of any other three leads, and the prediction residuals were used to assess the quality of the ECG. In addition to the residuals, they also included information about the amplitude variation of the ECG, achieving an accuracy of 0.90 in event 1. Moody [11] defined three simple heuristic rules based on ECG amplitude criteria. This algorithm attained a score of 0.896 in event 1. Johannesen et al. [13, 20] proposed a threshold detector based on ECG amplitude metrics (saturation, flat baseline) and the quantification of the noise content of the ECG which scored 0.88 in event 1. Langley et al. [12] used basic ECG amplitude metrics to develop an algorithm yielding an score of 0.868 in event 1. This work was later improved [19] by including QRS quality metrics and noise characterization achieving an accuracy of 0.914. Chudacek et al. [14] used five simple rules based on common ECG measurements (flat baseline, amplitude, baseline drift). They scored 0.828 in event 1 and a remarkable 0.872 in event 3 (2nd place).

Although the cross-correlation among leads has been used as a single metric to classify the quality of the ECG, the structure of the covariance matrix of the ECG signal leads has not been explored in the scientific literature. The 12-lead ECG signals are different projections of the same electrical activation process of the heart, and consequently the covariance matrix of the leads should have a particular structure. Cross-covariance of signals has been widely used in other signal processing applications, such as spectral estimation, antenna beamforming, equalization or pattern recognition, among many others. Also, it has been successfully used in ECG signal processing [24, 25], including data compression and filtering [26], ST-T segment analysis [27], and ventricular repolarization analysis [28].

The objective of this work is to provide a novel technique to classify the quality of the ECG signal based on the covariance matrix of the leads using a simple and computationally low-cost algorithm. Eigenvalues of the covariance matrix are fed into three different supervised binary classifiers: two tree inducers, namely CART [29], and C4.5 [30], and a propositional rule learner, namely RIPPER [31]. These algorithms are simple and provide useful interpretable models for the classification process, thus allowing us to gain better understanding about the relationship between data and the classification outcomes. To analyze the performance of the proposed methodology, we used the PhysioNet/CinC Challenge 2011 data [2], so the presented results can be compared to the work of challenge participants (Table 1) using the same database.

## Methods

### ECG collection

We used the PhysioNet/CinC Challenge 2011 data [2], which comprise a collection of standard 12-lead ECG recordings (leads I, II, III, aVR, aVL, aVF, V1, V2, V3, V4, V5, and V6) with full diagnostic bandwidth (0.05 through 100 Hz). The recordings were collected using conventional ECG machines, instead of using the equipment originally planned, which was intended to replicate the conditions to record and transmit ECG from rural patients for their remote analysis [32]. The leads are recorded simultaneously for 10 seconds; each lead is sampled at 500 Hz with 16-bit resolution. These signals were manually annotated by a group of 23 volunteers, giving each ECG a reference quality classification of Acceptable (*AC*) or Unacceptable (*UN*). ECGs signals are publicly available for download at Physionet database [32].

Note that ECG quality classification is not based on an objective quality metric, such as the Signal-to-Noise-Ratio, the percentage of detectable QRS waves, or the dynamic range among other possibilities. Instead, the annotated ECG quality classification was the result of a combination of subjective criteria that somehow tried to estimate the usability for clinical purposes of a given ECG. Volunteer annotators, having different levels of clinical knowledge, graded each ECG on a five letter scale: A (an outstanding recording with no visible noise or artifact; B (a good recording with transient artifact or low level noise that does not interfere with interpretation; all leads recorded well); C (an adequate recording that can be interpreted with confidence despite visible and obvious flaws, but no missing signals); D (a poor recording that may be interpretable with difficulty, or an otherwise good recording with one or more missing signals); or F (an unacceptably poor recording that cannot be interpreted with confidence because of significant technical flaws). Letter grades were mapped to numerical values (A = 0.95, B = 0.85, C = 0.75, D = 0.6, and F = 0), and scores from different annotators were averaged to a final value. An ECG was classified as *AC* if two or more grades were available, the average grade larger than 0.7, and no more than one grade was F. ECGs having an average lower than 0.7, were labeled *UN*.

Each of the ECGs available for the Challenge was randomly assigned to one of three groups: the training set A (Dataset A) containing 998 ECGs; the test set B (Dataset B) containing 500 ECGs used in events 1 and 2 of the Challenge [2], for which classification labels were withheld; and set C (Dataset C) containing 500 ECGs used in event 3 but not available to challenge participants.

### ECG signal model

According to the Volume Conductor Theory [33], the ECG signal $x_l(t)$ recorded by lead *l* can be approximated by the sum of the bioelectric signals $s_i(t)$ generated by the myocardium that travel through the volume conductor under quasi-static conditions (instantaneous propagation). Thus, the signal $x_l(t)$ can be modeled as

1 $$\begin{aligned} x_l(t)=\sum _{i=1}^N a_{li} s_i(t), \end{aligned}$$

where $a_{li}$ is the attenuation suffered by the bioelectric component *i* when it propagates through the volume conductor to reach lead *l* [34]. Thus, the morphology of the signals recorded from different leads will differ due to the factor $a_{li}$. However, since $x_l(t)$ measure the same bioelectric phenomena they should be statistically correlated in absence of noise and/or artifacts.

The model described in (1) can be extended to consider *L* simultaneous leads as [35]

2 $$\begin{aligned} \mathbf x(t) = \mathbf A \mathbf s(t), \end{aligned}$$

where $\mathbf x(t) = \left[ x_1(t),x_2(t),\ldots ,x_L(t)\right] ^T$ is the $L\times 1$ vector of ECG signals, $\mathbf s(t) = \left[ s_1(t),s_2(t),\ldots ,s_N(t)\right] ^T$ represents the *N* bioelectric sources at the heart, and $\mathbf A$ is the $L\times N$ matrix which can be expressed as

3 $$\begin{aligned} \mathbf A = \left[ \begin{array}{cccc} \mathbf a_1&\mathbf a_2&\cdots&\mathbf a_N \end{array} \right] , \end{aligned}$$

where the columns of $\mathbf A$ correspond to the attenuation suffered by bioelectric component *i* when propagating through the volume conductor to reach lead *l* [34]: $\mathbf a_i= \left[ a_{i1}, a_{i2}, \ldots , a_{iL} \right] ^T$.

In the presence of noise and/or artifacts (2) becomes

4 $$\begin{aligned} \mathbf x(t) = \mathbf A \mathbf s(t) + \mathbf n(t), \end{aligned}$$

where $\mathbf n(t)=\left[ n_1(t),n_2(t),\ldots ,n_L(t)\right] ^T$ is the $L \times 1$ vector containing the noise contribution for each lead.

Lead signals in a standard 12-lead ECG are computed in ECG equipment by linear combination of 9 signals captured by electrodes, so the rank of any data structure built from the 12 leads cannot be larger than 9. Moreover, the bipolar lead signals are constructed by using as reference a specific lead signal (for limb leads) or a combination of them (for precordial leads). Thus, the bipolar configuration of lead signals imposes the rank of the data structure defined on (2) to be equal or lower than 8. Consequently we have considered $L=8$. We checked (simulations not shown) that the inclusion of the signals from leads III, aVR, aVL and aVF did not add any useful information to ECG quality estimation, but just added low level quantification noise generated internally by the ECG equipment.

### Lead covariance

According to the signal model (2), and assuming zero-mean ECG signals and the absence of noise, the covariance matrix among the signals $\mathbf x(t)$ can be obtained as

5 $$\begin{aligned} \mathbf R_x = E \left[ \mathbf x(t) \mathbf x^T(t) \right] = E \left[ \mathbf A \mathbf s(t) \mathbf s^T(t) \mathbf A^T \right] = \mathbf A \mathbf R_s \mathbf A^T, \end{aligned}$$

where $E[\cdot ]$ stands for expected value operator, and where $\mathbf R_s$ is the covariance matrix of bioelectric sources at the heart. Since we have assumed there are *N* independent sources, the rank of the matrix $\mathbf R_s$ is necessarily *N*. Therefore, the eigendecomposition of matrix $\mathbf R_x$ would provide *N* non-zero eigenvalues.

In the case of the signal model (4), in the presence of noise and artifacts the covariance matrix of $\mathbf x(t)$ becomes

6 $$\begin{aligned} \mathbf R_x & {} = E \left[ \mathbf x(t) \mathbf x^T(t) \right] \nonumber \\ & {} = E \left[ \left( \mathbf A \mathbf s(t) + \mathbf n(t) \right) \left( \mathbf s^T(t) \mathbf A^T + \mathbf n^T(t) \right) \right] \nonumber \\ & {} = \mathbf A \mathbf R_s \mathbf A^T + \mathbf Q_n, \end{aligned}$$

where $\mathbf Q_n$ is the matrix containing the power received at each lead due to noise and artifacts (i.e., components coming from sources as 50/60 Hz power leakage, muscular artifacts, movements of the electrodes...). Consequently, in this case $\mathbf R_x$ will have $8-N$ eigenvalues describing noise and artifacts, and *N* eigenvalues corresponding to the heart electrical signals. Also, and in the absence of noise three eigenvalues are expected to be high [36]. Note that in (6), we have assumed that the signal and the noise spaces are uncorrelated. Therefore, this model can be used under different noise source conditions, such as white noise corruption, missing leads (or saturation effects) or signal artifacts. As a machine learning approach is followed, $\mathbf Q_n$ estimation and separation of signal and noise spaces are not required but replaced by the learning of the mapping of $\mathbf R_x$ eigenvalue distribution to the labels given by the experts.

Taking into account the aforementioned considerations, the quality of an ECG can be estimated by analyzing the covariance matrix structure of the 8-leads ECG. Assuming ergodicity, covariance matrix $\mathbf R_x$ can be easily estimated from the samples. In contrast to other signal processing applications, note that generally some leads are much noisier than others, and artifacts variability is large, consequently, we cannot assume a simplistic statistical model for them. So standard statistical hypothesis tests to detect the number of “signal eigenvalues” from a sample covariance matrix (AIC, MDL) [37–39] are not applicable for this scenario because they assume an a priori stochastic model for the noise and artifacts. Specifically those algorithms assume an i.i.d. (independent and identically distributed) model for noise and/or interference, which does not apply for most common degradation causes for the ECG lead signals. Thus, we propose the use of supervised machine learning algorithms to classify the quality of ECGs from the eigenvalues of their covariance matrix, as follows:

1. Form the X-Lead ECG data matrix, $\mathbf X$, by stacking up the 8 rows containing *K* time-samples of the 8 leads.
2. Estimate the sample covariance matrix $\hat{\mathbf {R}}_x= {1 \over K} \mathbf X \mathbf X^T$.
3. Compute the eigenvalues, $\lambda _i$ from $\hat{\mathbf {R}}_x$, for $i=1, 2,\ldots , 8$.

During these computations and for the classification process, neither signals nor eigenvalues were scaled or normalized.

### Machine learning algorithm

Given a training dataset $\mathcal {D}=\{(\mathbf {x}_i,y_i)\}_{i=1}^M$, where $\mathbf {x}_i=[\lambda _{1}^i,\ldots , \lambda _8^i]^T \in \mathbb {R}^8$ are the predictors of the *i*-th record and $y_i \in \{AC, UN\}$ denote its label, supervised binary classification [40] considers the selection of a model *f* in a space $\mathcal {H}$ that minimizes a criterion (usually the classification error) on $\mathcal {D}$ and provides good generalization (i.e., good performance on unseen data). Among the available methods to design classifiers, exploratory-data-analysis classification procedures were selected to give both an insight in data structure and to provide easy-to-interpret models. Namely, we selected two tree inducers, CART [29] and C4.5 [30], and a propositional rule learner, RIPPER [31]. While simple, these algorithms provide useful interpretable models for the classification process, allowing us to better understand the goodness and limitations of our approach regarding the available data. We have also included a SVM [41] classifier with a radial basis function kernel with the aim of comparing the above methods with a state-of-the-art classifier.

Best machine learning practices were followed. Dataset A was divided into training and test blocks, where the test block was used once to provide an estimate of the accuracy. Model selection was done only on the training block. As data is scarce, 10-fold cross-validation was performed to estimate on Dataset A the accuracy, sensitivity (proportion of *UN* records classified as *UN*), specificity (proportion of *AC*  records classified as *AC*) and the area under the receiver operating characteristics curve (AUC) for each classifier. Then, the best model was trained on the complete Dataset A and evaluated on Dataset B. Labels of Dataset B were not available and its predicted labels were sent to the Challenge organizers to get their classification accuracy.

## Results

The behavior of the proposed classifiers has been analyzed by using the Dataset A, since this is the only data that provide both the ECG signals and their corresponding labels (*AC*, *UN*). First, we have carried out an exploratory analysis using the Dataset A. Then, we have estimated the performance of the proposed classifiers through cross-validation using the Dataset A. Finally, we have tested our best classifier on the Dataset B (whose labels are withheld by PhysioNet/CinC Challenge 2011 organisers).

### Exploratory analysis: analysis of eigenvalues and classification

**Figure 1:**
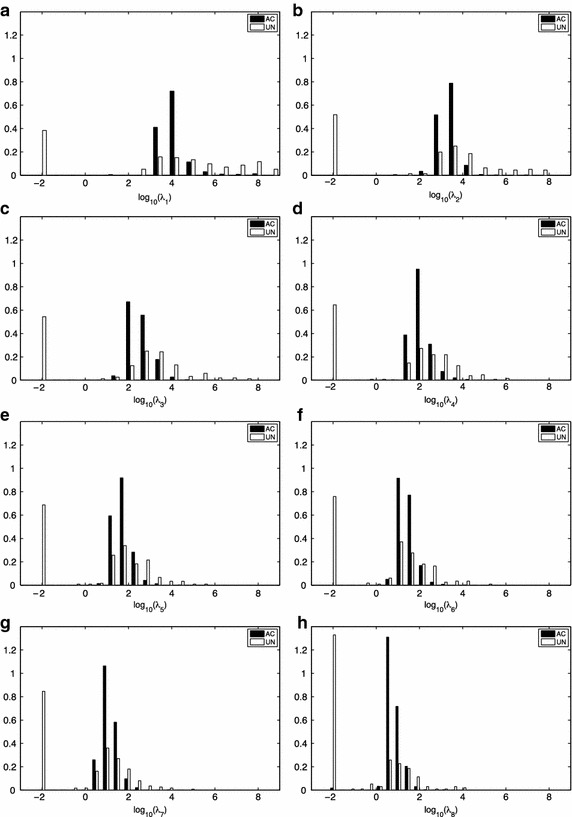
Eigenvalues analysis. Normalized histograms (**a**–**h**) for the eigenvalues of Dataset A divided by class. $\lambda _i \le 10^{-2}$ were grouped in the $10^{-2}$ bin.

The normalized (unit area) histograms of the eigenvalues for the *AC* and *UN* ECG records of the Dataset A are shown in Figure 1. A general pattern is observed in all histograms, which is best exemplified in $\lambda _8$ (Figure 1h). There is a proportion of *UN*  records that have smaller eigenvalues than the *AC*  ones ($\log _{10} (\lambda _8) \le -2$). Also, there is a proportion of *UN*  records that have higher eigenvalues than the *AC*  ones ($\log _{10} (\lambda _8) \ge 2$).

Classifiers were induced with the WEKA [42] package using Dataset A. Note that no filtering of ECG signals was performed (except for mean subtraction) to compute the eigenvalues. A number of preprocessing algorithms (noise filtering) were evaluated [1, 10], showing no improvement in the classification performance.

#### CART classifier

**Figure 2:**
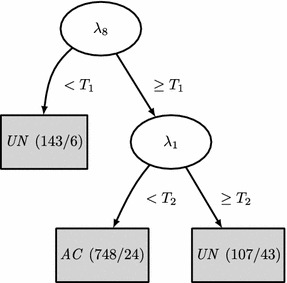
Tree induced by CART. The* numbers* associated to the label, e.g. $UN\,\,(143/6)$, indicate the number of registers classified by the rule and the number of errors it made evaluating the algorithm on Dataset A, respectively. $\log _{10}(T_1) = 0.145$, $\log _{10}(T_2) = 5.08$.

**Figure 3:**
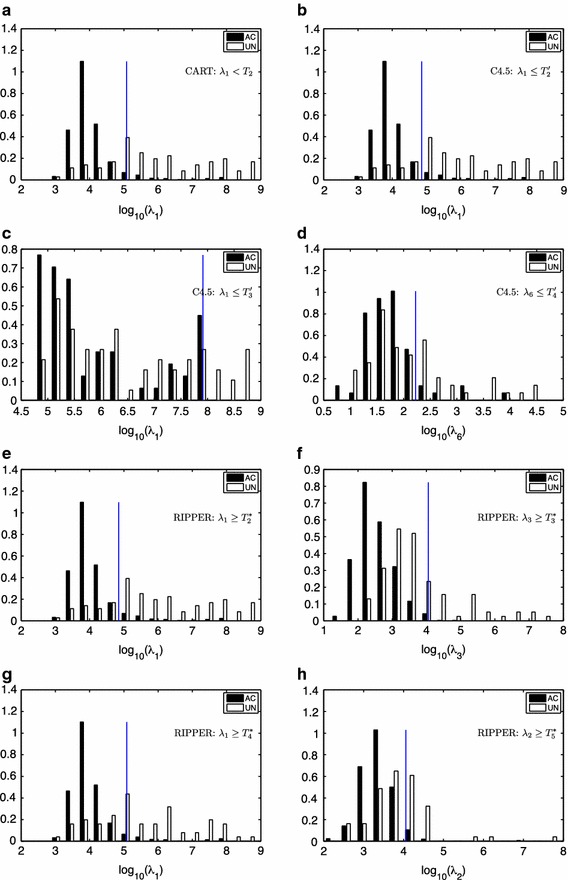
Normalized histograms (**a**–**h**) of the eigenvalues to be classified by a rule. $\lambda _i \le 10^{-2}$ were grouped in the $10^{-2}$ bin. The* vertical line* represents the threshold for the associated rule.

Figure 2 shows the tree induced by CART, which is a very simple classifier that only looks at the highest ($\lambda _1$) and lowest ($\lambda _8$) eigenvalues. If $\lambda _8 < T_1$, the record was considered *UN*. This rule classified 143 records with only 6 mistakes in Dataset A. The remaining 855 records were classified by using the information contained in $\lambda _1$. If $\lambda _1\ge T_2$, the record was considered *UN*. This rule correctly classified 788 records and made 67 mistakes in Dataset A, note that the results reported here correspond to train and test on the same Dataset A. Performance evaluation using cross-validation is reported in the next subsection. The histogram of $\lambda _1$ for the ECGs classified by this rule is show n in Figure 3a, where it can be seen that most *AC*  cases had $3\le \log _{10}(\lambda _1)\le 5$.

#### C4.5 classifier

**Figure 4:**
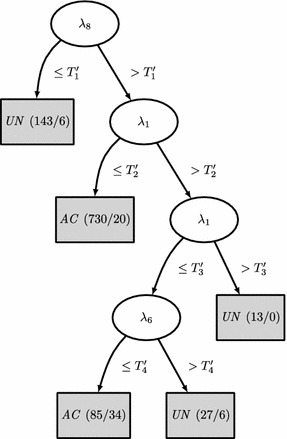
Tree induced by C4.5. $\log _{10}(T_1') = 0.0870$, $\log _{10}(T_2') = 4.86$, $\log _{10}(T_3') = 7.91$ and $\log _{10}(T_4') = 2.23$.

**Figure 5:**
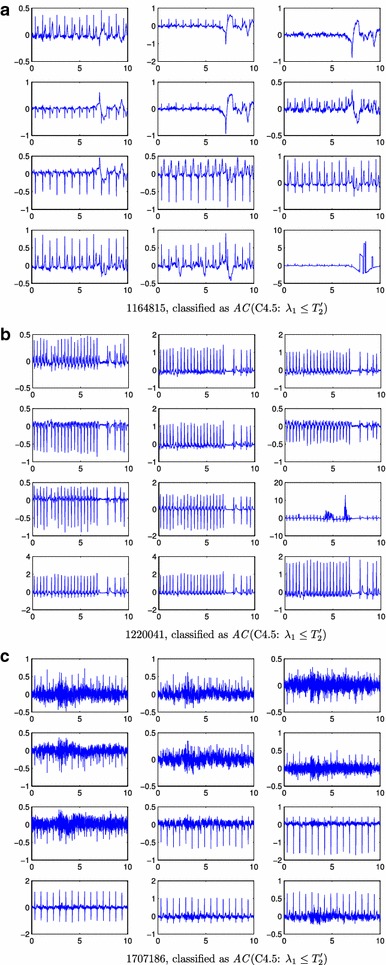
*UN* ECG records wrongly classified by the classifiers (**a**–**c**). This corresponds to ECG signals labelled as *UN* that have been classified as *AC* by the algorithms. For each* panel*, it is indicated the record-id, the name of the algorithm, and the rule responsible for the misclassification. x-axes units are in seconds and y-axes are mV.

**Figure 6:**
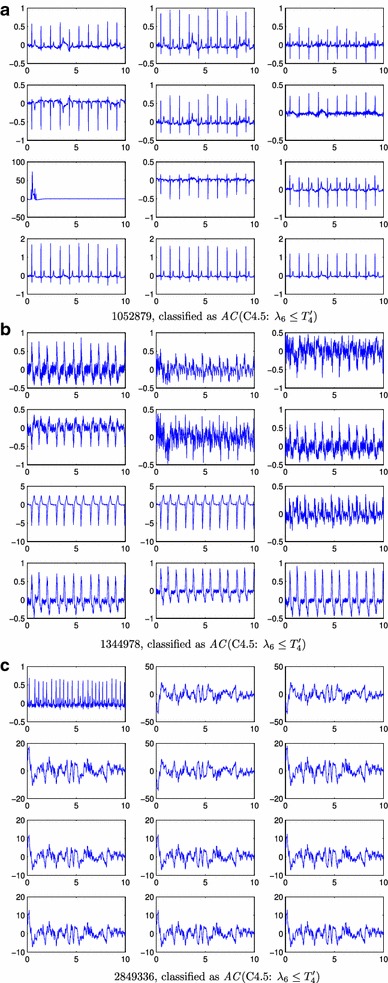
*UN* ECG records wrongly classified by the classifiers (**a**–**c**). This corresponds to ECG signals labelled as *UN* that have been classified as *AC* by the algorithms. For each* panel*, it is indicated the record-id, the name of the algorithm, and the rule responsible for the misclassification. x-axes units are in seconds and y-axes are mV.

**Figure 7:**
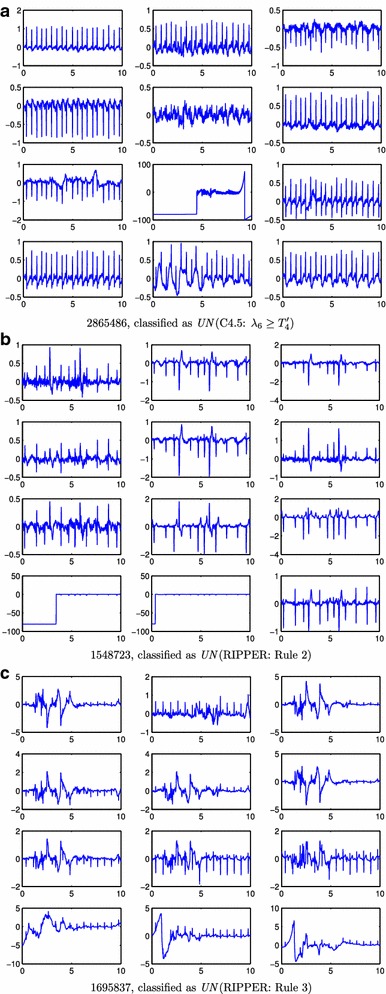
*AC* ECG records wrongly classified by the classifiers (**a**–**c**). This corresponds to ECG signals labelled as *AC* that have been classified as *UN* by the algorithms. For each* panel*, it is indicated the record-id, the name of the algorithm, and the rule responsible for the misclassification. x-axes units are in seconds and y-axes are mV.

Figure 4 shows the tree induced by C4.5. Compared to CART, this classifier further refines the records that have a signal space with 8 components (a threshold on $\lambda _8$ is the first rule), and a high value for $\lambda _1$. This refinement firstly classifies as *AC*  the records that fulfilled $\lambda _1 \le T_2'$ (730 records, 20 errors) with $T_2' < T_2$, as it is shown in Figure 3b. This rule incorrectly classified as *AC*  several ECG records with moderate artifacts and a low power of high frequency noise (see Figure 5a–c). For the remaining records $\lambda _1$ is thresholded again ($T_3'$) to rule out the 13 *UN*  records with higher eigenvalues compared to the *AC*  records (see Figure 3c), showing no mistake. The final rule classified the remaining 112 ECGs (see their eigenvalues histograms in Figure 3d) using a threshold on $\lambda _6$. Most of the 34 ECGs incorrectly classified by this rule as *AC*  were records with an artifact and high frequency noise (see Figure 6a). Other incorrect *AC*  classifications were records with several noise contributions that had similar signal spaces to acceptable records (see Figure 6b) or records without a clear QRS pattern (see Figure 6c). There were only 6 ECG records incorrectly classified as *UN*  and all of them shared a high power interference and several noise contributions (see Figure 7a).

#### RIPPER classifier

Table 2 shows the rules induced by this classifier. The classification label is assigned according to the first rule that is satisfied. This classifier firstly focuses, as the previous ones, on the subspace distribution of the records: if $\lambda _8 \le T_1^*$ the record is declared *UN*. Then, it considers the subspace of $\{\lambda _1, \lambda _2, \lambda _3\}$ to check two abnormal situations: (i) whether both $\lambda _1$ and $\lambda _3$ are above two thresholds (see Figure 3e, f), which has 13% error; and (ii) whether $\lambda _1$ and $\lambda _2$ are both above two thresholds (see Figure 3g, h), which has 40% error.

**Figure 8:**
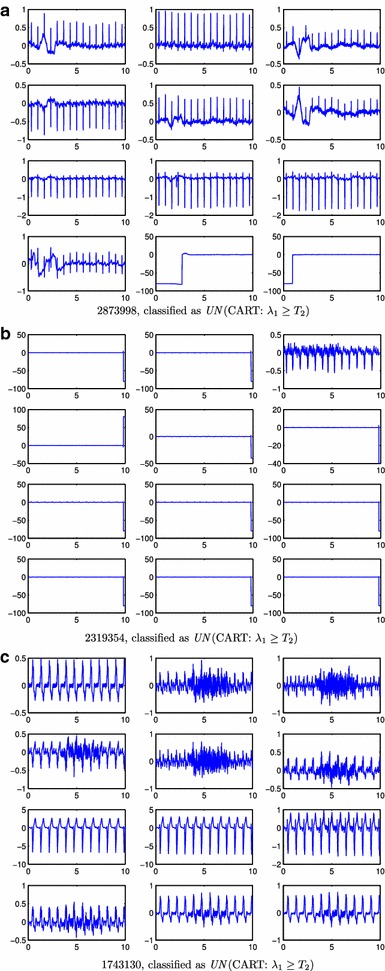
*AC* ECG records wrongly classified by the classifiers (**a**–**c**). This corresponds to ECG signals labelled as *AC* that have been classified as *UN* by the algorithms. For each* panel*, it is indicated the record-id, the name of the algorithm, and the rule responsible for the misclassification. x-axes units are in seconds and y-axes are mV.

#### Classification performance

- Cross-validation on Dataset A: 10-fold cross-validation results on Dataset A are shown in Table 3. AUC was obtained with the predictions on each test-fold. If we consider accuracy, RIPPER performs the best in this dataset, if we consider AUC, C4.5 is the best and CART provides the most balanced result considering specificity and sensitivity. There is therefore no clear cut ranking for these classifiers as they were induced to maximize accuracy.
- Testing on Dataset B: Considering the RIPPER our best classifier (in terms of accuracy), the predicted labels obtained by this classifier in Dataset B were sent to the organizer of the PhysioNet/CinC Challenge 2011 and obtained an accuracy of 0.898 (449 out of 500).

The complexity of the algorithm is the sum of the complexities of the calculation of the covariance matrix ($$8*L*K^2$$ operations, see BLAS dgemm routine), the calculation of the eigenvalues of the covariance matrix (O($$L^3$$) operations, see LAPACK dsyev routine) and the complexity of the classifier, which in the case of the above RIPPER classifier is only 5 comparisons and 2 logical operations, which is extremely fast [43, 44].

## Conclusions

This paper presents a classification approach that combines linear signal subspace analysis (the eigenvalues of the covariance matrix) with interpretable machine learning. One of the strengths of the proposed approach is the interpretability of the results, which provide further insight into the ECG quality estimation problem and the shortcomings of our proposal.

The analysis of the eigenvalues of the Dataset A, shown in Figure 1, revealed that *UN*  records have either simpler or richer signal spaces than the *AC*  ones. That is, eigenvalues for *UN*  records showed a bimodal distribution, having either small ($\log _{10}(\lambda _i)\le - 2$) or large ($\log _{10}(\lambda _i)\ge 2$) eigenvalues, while for *AC*  records the distribution of eigenvalues were more concentrated around their mean. The simpler signal space can be attributed to poorly connected or disconnected leads while the richer signal space is probably caused by external noise sources and artifacts. Given these differences in the eigenvalue distribution for *UN*  and *AC*  records, we also evaluated the condition number, $\kappa =\frac{\lambda _1}{\lambda _8}$, of the sample covariance as a possible feature for classification. However, its introduction provided no improvement in performance over the set of eigenvalues because our classifiers looked for the best thresholds for each feature, which is equivalent to look for the best threshold on a quotient of two of the eigenvalues.

The classifiers decision rules and their errors also revealed new details of the classification problem and how our proposed methodology addressed it. The CART classifier (Figure 2) demonstrated a high accuracy, deciding on its first rule on the lowest eigenvalue ($\lambda _8$). This provides evidence suggesting that when a non-full-rank covariance matrix ($\lambda _8 < T_1$) is obtained there might have been some problems with the lead connections. In fact, the 6 *AC*  ECGs that were wrongly classified as *UN*  by this rule had one of the precordial leads to zero or to a constant value. The second CART rule decided on $\lambda _1$, that for most *AC*  cases is in the range $3\le \log _{10}(\lambda _1)\le 5$. Many *UN*  cases had larger $\lambda _1$ values, which can be attributed to: (i) high noise or high power artifacts present on the record; and (ii) $\lambda _1$ captures most energy, meaning that record is not rich enough or that the same noise/artifact is present in several leads (specially if it has high power). Figure 8a–c show three *AC*  records classified as *UN*  by this rule. Figure 8a represents one example of two high power artifacts in two leads. These artifacts dominate the energy of the signals and they contribute most to the first two eigenvalues. Figure 8b shows a common artifact that affects all but one lead, therefore the first eigenvalue is high as this single artifact dominates the energy of the ECG. However, classification error in Figure 8b may be an example of a mistake in the challenge database annotations, since only one lead contains usable information. Figure 8c shows a case with high frequency noise affecting several leads. This noise mainly affects the second eigenvalue. Also in this record, some of the leads have 5 times higher voltage than other leads, which is likely the cause of misclassification. Besides this, the CART classifier also presents other limitations: high energy artifacts and/or wandering baseline in some leads can make the classifier to wrongly reject a record classified as *AC*. Nevertheless, these limitations can be overcome, in part, by baseline removal and clipping, at the expense of more computational processing requirements.

With respect to CART, the C4.5 classifier produced a refinement for cases with large $\lambda _1$. Figure 5a–c show some *UN*  records wrongly classified as *AC*  by C4.5. Whether noise or artifacts made the record labeled as *AC*  or *UN*  is unclear to the authors (the reader can compare the ECGs presented in Figures 5, 6, 7 and 8). On the other hand, the rule $\lambda _1 \le T_2'$ showed another limitation of the proposed algorithm. Some *UN*  records with low power noise were not easy to separate from *AC*  records by just considering the power distribution of the different components of the signal space (see Figure 5c). The last rule for C4.5 relied on $\lambda _6$. The rationale behind this last rule is again related to dimensional spaces. In a “perfectly clean” ECG, three eigenvalues are expected to be high (the ones related with the main components of the heartbeat), while the rest of the eigenvalues should be smaller. Therefore, if $\lambda _6$ is above a threshold, this represents that there is too much energy out of the three main eigenvalues, which indicates several unexpected sources of noise. The sixth eigenvalue is selected for the last decision because the classifier learned from the training Dataset A the amount of perturbation (other signal/noise/interference sources) that is allowed to classify a record as *AC*. The limitations of the C4.5 classifier, e.g. *UN*  records with several noise contributions that have similar signal spaces that *AC*  records or records without a clear QRS pattern, could be alleviated with more processing by rhythm-analysis.

Finally, the RIPPER classifier did not show types of errors different from those already presented for CART and C4.5. Most *AC* records wrongly classified by Rules 2 and 3 of RIPPER (Table 2) were records with artifacts, high frequency noise and base line wandering (see Figure 7b, c for an example of a record misclassified by Rules 2 and 3, respectively).

**Table 2:** RIPPER ruleset, where $\log _{10}(T_1^*) = 0.0863$, $\log _{10}(T_2^*) = 4.85$, $\log _{10}(T_3^*) = 4.06$, $\log _{10}(T_4^*) = 5.09$ and $\log _{10}(T_5^*) = 3.75$

1	($\lambda _8 \le T_1^*$) $\Rightarrow$ Label = *UN* (143/6)
2	($\lambda _1 \ge T_2^*$) $\wedge$ ($\lambda _3 \ge T_3^*$) $\Rightarrow$ Label = *UN* (30/4)
3	($\lambda _1 \ge T_4^*$) $\wedge$ ($\lambda _2 \ge T_5^*$) $\Rightarrow$ Label = *UN* (47/19)
4	Label = *AC* (778/34)

The first rule that fires decides the label. Eigenvalues are in $\log _{10}$ scale.

**Table 3:** Performance comparison of classifiers on Dataset A using 10-fold cross-validation. Alg., Acc., Sens., Spec. and AUC stand for algorithm, accuracy, sensitivity, specificity, and area under the ROC curve, respectively

Alg.	Acc. (%)	Sens. (%)	Spec. (%)	AUC
CART	92.1	84.4	94.3	0.913
C4.5	91.7	77.8	95.7	0.925
RIPPER	92.7	83.1	95.5	0.910
SVM	92.5	70.7	99.0	0.902

As previously stated, most of the above mentioned shortcomings of the proposed classifiers could be mitigated in part by introducing additional steps to conveniently process the ECG signals. This, however would increase the computational cost of the algorithm. Moreover, labeling of some of the ECGs of Challenge database are borderline or errors. When analyzed in detail the errors of classifiers proposed herein, particularly those shown in Figures 5, 6, 7 and 8, it seems that our classifiers fail precisely in those borderline cases or those that are clear labeling errors. Also, our approach works on multi-lead recordings, while other studies developed methods suitable for single-lead recordings [4, 8, 16, 17].

Here, we present a simple, fast and reliable approach for ECG quality estimation that combines linear signal subspace analysis with machine learning. On the one hand, linear subspace analysis estimates the energy of the different ECG components; on the other hand, interpretable machine learning discovers how experts classify and provides simple and easy to understand decision rules. The understanding gained with the proposed approach, on how ECG quality is estimated by cardiologists, could be useful to design different algorithms. The result is a new ECG quality classifier with extremely low computational burden and, if we had submitted this classifier to the open source PhysioNet/CinC Challenge 2011, this classifier would have ranked second best (event 2, Table 1). Therefore, the proposed approach is particularly suitable for inexpensive portable ECG monitoring systems. A Java code implementing this approach can be found at https://github.com/obarquero/ECG_quality.
